# A Novel Method of Synthesizing Polymeric Aluminum Ferric Sulfate Flocculant and Preparing Red Mud-Based Ceramsite

**DOI:** 10.3390/ma17061239

**Published:** 2024-03-07

**Authors:** Zhilei Zhen, Chenxi He, Yanrong Wang, Haotian Ma

**Affiliations:** College of Urban and Rural Construction, Shanxi Agricultural University, Taigu 030801, China; zhencheng@sxau.edu.cn (Z.Z.); 17836509636@163.com (C.H.); wyr@sxau.edu.cn (Y.W.)

**Keywords:** red mud, acid leaching, polymeric aluminum ferric sulfate, phosphate

## Abstract

A synthetic flocculant of aluminum (Al) and iron (Fe) extracted from red mud (RM) has been widely used in sewage treatment, while the remaining RM residue has been ignored. This study aimed to synthesize polymeric aluminum ferric sulfate (PAFS) flocculant from RM by acid leaching and then use the acidified RM residue to produce an acid RM-based ceramsite (ARMC) by mixing bentonite, hydroxypropyl methylcellulose, and starch. Our results showed that sintering, reaction temperature, H_2_SO_4_ concentration, reaction time, and liquid-to-solid ratio had an obvious effect on the leaching of Al and Fe in RM, which was a necessary prerequisite for the efficient PAFS flocculants. At a PAFS dosage of 60 mg/L, turbidity and phosphate removal rates were 95.21 ± 0.64% and 89.17 ± 0.52%, respectively. When the pH value was 8.0, the turbidity and phosphate removal efficiency were 99.22 ± 0.66% and 95.98 ± 1.63%, respectively. Considering the adsorption capacity and mechanical properties, the best conditions for ARMC production included using 60% ARM and ceramsite calcination at 600 °C, with the BET surface area 56.16 m^2^/g and a pore volume of 0.167 cm^3^/g. Thermogravimetric analysis indicated that 400 °C was a reasonable preheating temperature to enhance the ARMC mechanical strength, as this temperature allows the removal of surface-adsorbed and constituent water. Under a scanning electron microscope, the ARMC appeared rough before adsorption, while relatively uniform pores occupied it after adsorption. Our conclusion will help to improve the zero-waste strategy of RM and speed up the industrial production of RM in flocculants as well as utilizing ARMC as a new type of adsorbent for phosphorus adsorption in sewage treatment.

## 1. Introduction

Red mud (RM) is a strong alkaline industrial solid waste produced during alumina production from bauxite [1]. Depending on the iron oxide content, RM can be red, reddish-brown, dark-red, or gray. Moreover, it has a high-water content and contains harmful components, such as heavy metals. Currently, 1.5–2.0 t of RM is produced from 1 t of alumina. According to the National Bureau of Statistics, China’s alumina production increased by 5% from that recorded in 2020 to 777, 475,000 t in 2021, and a plan is underway for increased production. Therefore, in the near future, the environmental issues associated with the massive accumulation of RM will become severe [2,3], endangering the survival of animals and plants. The Chinese government pays close attention to RM-related challenges and actively promotes the comprehensive utilization of RM.

RM mainly comprises fine particles, such as aluminum (Al), iron (Fe), silicon (Si), titanium (Ti), sodium (Na), and rare earth elements [4]. The recovery of metals from RM has become the focus of research. The recovery rates of Fe through reduction roasting with pulverized coal using hydrocyclone and a two-stage magnetic separation process were more than 70% and more than 75%, respectively [5]. Phosphoric acid was used to prepare active biochar from pitaya peel as a new material for the adsorption and recovery of scandium from RM leachate. The results showed that at a pH of 3, the recovery rate of scandium was 83% [6]. At 1323 K, Na can be effectively separated from Na-rich slag by super-gravity, and the recovery rate of Na from anorthite can reach 97.09% [7]. Numerous studies have explored the extraction of Al and Fe cations using hydrochloric or sulfuric acid to produce inorganic flocculants [8,9,10]. Owing to their high efficiency, non-toxicity, strong adaptability, and low price, inorganic polymer flocculants are widely used for sewage treatment [11,12,13]. Zhang et al. (2020) [14] obtained Fe-Al based flocculants by a microwave selective carbothermic reduction and magnetic separation technique. Cheng et al. (2021) [15] prepared a high-efficiency polyaluminum iron chloride flocculant by adjusting the pH of the leaching solution, the molar ratio of Al and Fe, and the polymerization temperature, and produced excellent flocculation performance. However, few studies consider how to use RM residue after metal extraction [16], which causes secondary environmental pollution.

Owing to its porous nature, RM can be transformed into a high-performance adsorbent. Activated RM has better adsorption capacity under bio-oxidation by the acidophilic bacterium [17], sintering at different temperatures [18], acid treatment [19], and the combination of acid treatment and heating [20]. Most studies on activated RM focused on the powdered form, impeding its practical application in wastewater treatment and resulting in serious issues, such as pore clogging, mass loss during operation, and difficulty in separating powdered absorbent. Recently, Ke et al. (2022) [16] tried to synthesize geopolymer from the iron-separated RM residue after treating the RM with carbothermal reduction for iron recovery, which achieved a zero-waste strategy to utilize RM. Meanwhile, the adsorption performance of geopolymer for pollutants has not been explored. Shabnam et al. (2019) [21] produced red-mud based ceramic and explored the application in a constructed wetland, and the result showed that the ceramic and *I. latifolia* enhanced the removal of phosphate. Therefore, ceramsite produced from acidified-RM (ARM) can become an ideal substrate for phosphorus removal in constructed wetlands [22]. Moreover, the ceramsite was sintered under optimum synthesis conditions and no environmental risks are caused by the release of heavy metals [23].

In our study, we aimed to suggest a novel method of producing flocculant from RM and using its subsequent residue to prepare ceramsite, explore the effects of phosphorus removal and turbidity reduction of the prepared flocculant, analyze the phosphorus removal performance and mechanical strength of acidified-RM ceramsite, and evaluate the acidified-RM ceramsite phosphate-adsorption mechanism.

## 2. Materials and Methods

### 2.1. Materials

**Figure 1:**
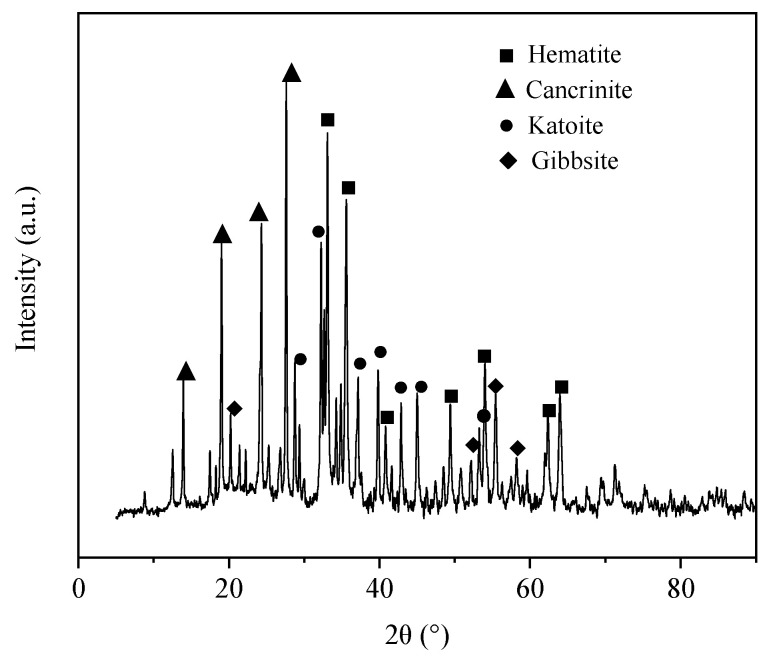
X-ray diffractometry patterns of raw red mud.

The main raw material, RM, was obtained from Shanxi Huaxing Aluminum, Lvliang, China and sieved over a 100-mesh. The composition of RM varies with the difference in natural bauxite deposits. The chemical composition of RM is presented in Table 1. RM comprised mainly 26.96%, 23.11%, and 20.25% of Fe_2_O_3_, Al_2_O_3_, and SiO_2_, respectively. X-ray diffractometry (XRD) showed that raw RM-specific chemical compositions were hematite, cancrinite, katoite, and gibbsite (Figure 1), consistent with the compositions presented in Table 1.

Chemical reagents, including H_2_SO_4_, NaOH, and H_2_O_2_, were obtained from the Shanghai Chemical Reagent Co., Ltd., Shanghai, China. The wastewater samples used for flocculant performance evaluation were obtained from the Taigu Wastewater Treatment Plant in Shanxi Province, Taigu, China (Table 2). Bentonite was purchased from the Tianjin Yandong Mineral Products Co., Ltd., Tianjin, China. Analytical grade hydroxypropyl methylcellulose (HPMC) was obtained from the Alfa Aesar (Tianjin) Chemical Co., Ltd. (Tianjin, China). Starch was selected as a pore-forming agent. All materials were comminuted, sieved through a 100-mesh screen (Xinxiang Dezhong Intelligent equipment Co., Ltd., Xinxiang, China), air-dried at 105 °C, and stored in a desiccator (Shenzhen Baiji Porpoise Biotechnology Co., Ltd., Shenzhen, China) for follow-up experiments. Phosphate solution was prepared with analytical grade potassium dihydrogen phosphate (K_2_HPO_4_, Sinopharm Chemical Reagent Co., Ltd., Shanghai, China).

### 2.2. Methods

#### 2.2.1. Red Mud Pre-Treatment Experiment

**Figure 2:**
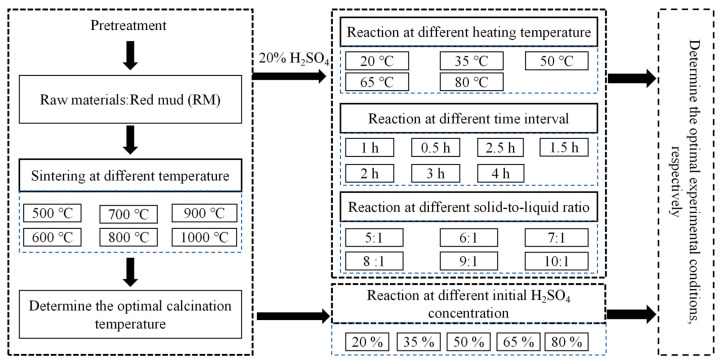
The process of determining the optional experimental conditions.

RM samples were sintered at different temperatures (500, 600, 700, 800, 900, and 1000 °C), and RM at optimal calcination temperature was determined (SRM) (Figure 2). Thereafter, the SRM was stirred at a speed of 300 rpm in a magnetic heating mixer (ZNCL-TS; Yuezhong Instrument Equipment Co., Ltd., Shanghai, China) under different H_2_SO_4_ concentrations (20, 35, 50, 65, and 80%), reaction temperatures (20, 35, 50, 65, and 80 °C), reaction times (0.5, 1.0, 1.5, 2.0, 2.5, 3.0, and 4.0 h), and solid–liquid ratios (5:1, 6:1, 7:1, 8:1, 9:1, and 10:1). Subsequently, acid leaching solution and residue were obtained by filtration with a vacuum suction filter (SHB-IIIA, Hangzhou Gengyu Instrument Co., Ltd., Hangzhou, China).

#### 2.2.2. Preparation of Polymeric Aluminum Ferric Sulfate

Under the best leaching conditions, Fe and Al oxides, including Al_2_(SO_4_)_3_, FeSO_4_, and Fe_2_(SO_4_)_3_, were formed by adding hydrogen peroxide (H_2_O_2_) to the solution and thereafter polymerized with an alkali to obtain PAFS (pH = 3.0–5.0). PAFS solution was stirred for 2 h at 60 °C and cooled to 25 °C. After 5 h of cooling, reddish-brown liquid PAFS was dried at 105 °C to obtain solid PAFS.

#### 2.2.3. The Synthesized Flocculant Performance Verification

The liquid 0.05% of PAFS was added to 100 mL of wastewater, stirred at 200 rpm for 2 min, and kept stable for 10 min. The turbidity and phosphate content of the supernatant were determined at different dosages (10, 20, 30, 40, 50, and 60 mg/L) and initial pH (2, 4, 6, 8, 10, and 12), and compared simultaneously with industrial polyaluminium chloride (PAC) (ZhengzhouTengquan Environmental Technology Co., Ltd., Zhengzhou, China).

#### 2.2.4. Preparation of Red Mud-Based Ceramsite

The RM residue after acid leaching (ARM), bentonite, HPMC, and starch were mixed to produce ceramsite. The mass ratio of bentonite was fixed at 20% to obtain a string of granular products. Based on the Riley phase diagram in the ceramic grain preparation method [24], the mass ratio of starch was fixed at 10% to guarantee a homogenous aerogenous spread. ARM and HPMC were mixed at a proportion of 70:0, 60:10, 50:20, and 40:30. An appropriate amount of deionized water (Molelement1830DF; Chongqing Molecular Water System Co., Ltd., Chongqing, China) was added to the mixture and stirred for 2 h at a speed of 100 rpm. Ball ceramsites with a diameter of 1 cm were made using abrasive tools and dried under natural conditions for 24 h. The ceramsites were preheated at 400 °C for 20 min and sintered at 500 °C to 1000 °C for 10 min in a muffle furnace (SX2-4-10N; Shanghai Yiheng Scientific Instrument Co., Ltd., Shanghai, China). ARMC was finally prepared for subsequent adsorption experiments.

#### 2.2.5. Phosphate Adsorption Capacities of ARMC

ARMC in Section 2.2.4 was added to 250 mL of phosphorus solution with a concentration of 100 mg/L and a liquid–solid ratio of 5:1. The rotating speed was 300 rpm for a certain time. The concentration of total phosphorus (TP) in the filtrate was determined by the ammonium molybdate phosphate spectrophotometry method [25].

#### 2.2.6. Fe and Al Analyses and Adsorbents Characterization

Fe and Al ions concentration of RM were determined by inductively coupled plasma mass spectrometry (Agilent, Santa Clara, CA, USA) and then calculated in the form of Al_2_O_3_ and Fe_2_O_3_. Turbidity was determined using a portable turbidimeter (Hach, Loveland, CO, USA). The phosphate concentration was determined by a spectrophotometer (UV1280; Biobase; Jinan, China). The chemical composition of RM was quantified using X-ray fluorescence (XRF, Axios mAx, PANalytical B.V., Almelo, The Netherlands). The mineralogical composition of RM, PAFS, ARM, and ARMC was characterized using an XRD (XD8 Advance, Bruker, Karlsruhe, Germany) with Cu Kα radiation at 40 kV and 30 mA, which was recorded in a 2θ range of 10–70° at a scan speed range of 0.02 deg/s. A Fourier transform infra-red (FT-IR) spectrophotometer (Nicolet NEXUS 670; ThermoFisher; Waltham, MA, USA) was used to determine the structural composition and characteristic functional groups of the PAFS. The specific surface area and pore size distribution of ARM-based ceramsites were determined using the Brunauer–Emmett–Teller (BET) nitrogen adsorption equilibrium method (Quantachrome; Autosirb-iQ; Boynton Beach, FL, USA). The microstructure of the ARMC was characterized using an X650 scanning electron microscope (SEM). Thermogravimetric analysis of ARMC was performed using a DTG-60 instrument (Autosorb-iQ-C chemisorption analyzer; Quantachrome; Boynton Beach, FL, USA) from 35 to 600 °C at a heating rate of 5 °C/min. Helium was used as a protective gas, and the flow rate was controlled at 20 mL/min. Mixed air was used as a purge gas, and the flow rate was controlled at 50 mL/min. The bulk density, apparent density, true density, and 1 h water adsorption capacity of ARMC were tested according to the Chinese standard (GB/T17431.2-2010) [26].

#### 2.2.7. Statistical Method

Three parallel sets of adsorption capacity ($q_{e}$) data were used for analysis. $q_{e}$ data are expressed as mean ± standard deviation (SD). The calculation formula for the phosphate adsorption capacity ($q_{e}$) of the ARMC is as follows:

(1) $$q_{e}=\frac{\left(C_{0}-C_{e}\right)}{m}$$

where $q_{e}$ is the adsorption capacity (mg/g); *m* is the ARMC quality (g); $C_{0}$ is the initial TP solution concentration (mg/L); $C_{e}$ is the TP solution concentration after adsorption equilibrium (mg/L).

Optimal leaching conditions were determined by measuring the leaching rates of Al and Fe from the solution. Three parallel sets of leaching rate (η) data were used for analysis. η data are expressed as mean ± standard deviation (SD). The leaching rate (*η*) of the ions was calculated using Equation (1):

(2) $$\eta =\frac{VC}{Q\alpha }\times 100\%$$

where *η* is the leaching rate of ions (%), *V* is the volume of the filtrate (mL), *C* is the concentration of the ions in the filtrate (g/mL), *Q* is the weight of RM (g), and *α* is the percentage of ions in the RM (%).

The bulk density (*ρ_b_*), apparent density (*ρ_a_*), actual density (*ρ_t_*), and 1 h water adsorption capacity of the samples were calculated using Equations (2), (3), (4), and (5), respectively.

(3) $$\rho_{b}=m_{1}/V_{1}$$

(4) $$\rho_{a}=m_{1}/\left(V_{2}-V_{W}\right)$$

(5) $$\rho_{t}=m_{1}/(V_{3}-V_{W})$$

(6) $$W_{a}=\frac{m_{2}-m_{1}}{m_{1}}\times 100\%$$

where *ρ_b_* is the bulk density (g/cm^3^), *m*_1_ is the mass of the dried samples (g), *V*_1_ is the stacking volume of samples (cm^3^), *ρ_a_* is the apparent density (g/cm^3^), *V*_2_ is the total volume of the sample and added water in the graduated cylinder (cm^3^), *V_w_* is the volume of the added water (cm^3^), *V*_3_ is the volume of the sample and added water after 1 h of water adsorption (cm^3^), *w_a_* is the water absorption capacity of the samples for 1 h (%), and *m*_2_ is the mass of the sample after water absorption (g).

The mechanical strength (MPa) of ARMC was determined using a microcomputer-controlled strength-testing machine (WE-300B; Jinan Zhongluchang Testing Machine Manufacturing Co., Ltd., Jinan, China) and calculated according to the following Equation (6) [27]:

(7) $$S=\frac{2.8P_{c}}{\pi X^{2}}$$

where *P_c_* is the crushing load (N) and *X* is the distance between the upper and lower loading plates (mm).

## 3. Results and Analysis

### 3.1. Effect of the Different Sintering Temperature on Red Mud Leaching of Al and Fe

**Figure 3:**
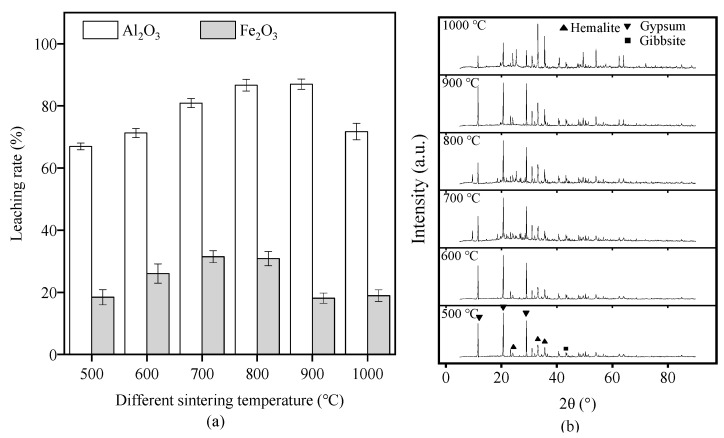
(**a**) The leaching rates of Al_2_O_3_ and Fe_2_O_3_ of raw red mud (RM) under different sintering temperatures. (**b**) X-ray diffractometry patterns of sintering red mud (SRM).

The effect of different sintering temperatures on RM leaching of Al_2_O_3_ and Fe_2_O_3_ was investigated at a 20% H_2_SO_4_, 20 °C reaction temperature, a 2 h reaction time, and a liquid-to-solid ratio of 10:1. The Al_2_O_3_ leaching rate initially increased continuously with the increasing sintering temperature, from 500 to 900 °C, and then significantly decreased at 1000 °C, whereas the Fe_2_O_3_ leaching rate increased as the sintering temperature increased, from 500 to 700 °C, and then began to decrease and became stable at 1000 °C (Figure 3a). An increased sintering temperature led to the loss of crystalline water from RM and the reduction of gibbsite (Al(OH)_3_) to Al_2_O_3_. The XRD pattern of SRM demonstrated a significant reduction in hematite and gibbsite peaks with the increasing sintering temperature (Figure 3b), indicating the leaching of Fe and Al ions due to the transformation of its crystalline structure. Therefore, through a comprehensive comparison of the leaching rates of Al and Fe and saving sintering energy consumption, the optimal sintering temperature was determined as 700 °C.

### 3.2. Effect of Reaction Temperature on Red Mud Leaching of Al and Fe

**Figure 4:**
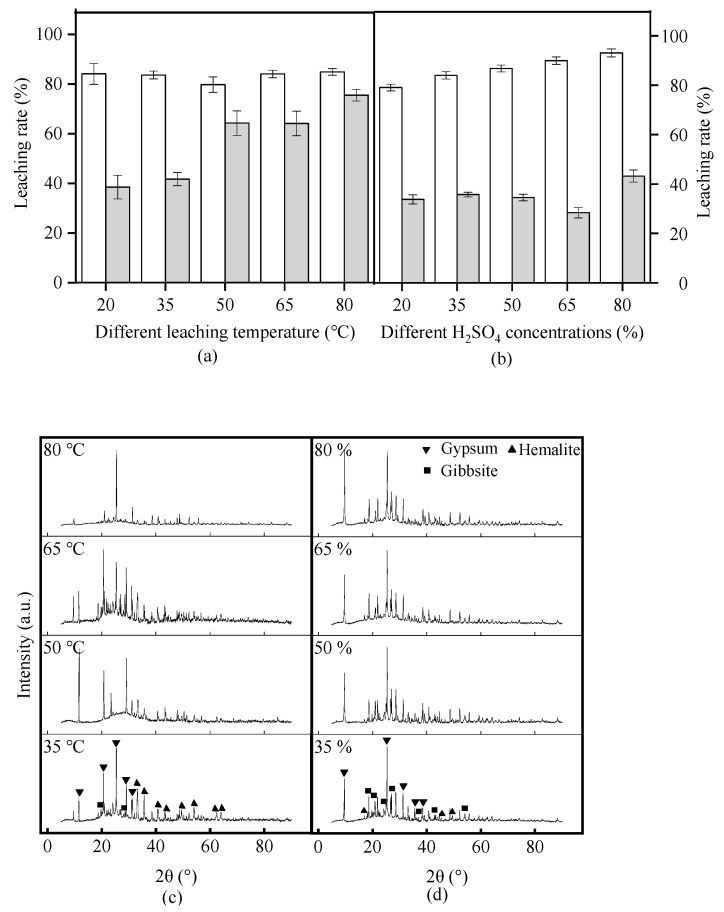
The leaching rates of Al_2_O_3_ and Fe_2_O_3_ of sintering red mud (SRM) under (**a**) different reaction temperature at constant: sintering temperature 500 °C, H_2_SO_4_ concentration 20 wt%, liquid–solid ratio 10:1 and reaction time 2 h; and (**b**) different H_2_SO_4_ concentration at constant: reaction temperature 20 °C, liquid-to-liquid ratio 10:1, and reaction time 2 h; and the X-ray diffractometry patterns of the acid leaching residue after different treatment condition: (**c**) reaction temperature at constant: sintering temperature 500 °C, H_2_SO_4_ concentration 20 wt%, liquid–solid ratio 10:1 and reaction time 2 h; and (**d**) H_2_SO_4_ concentration at constant: reaction temperature 20 °C, liquid-to-liquid ratio 10:1, and reaction time 2 h.

The SRM was added to a 20% concentrated H_2_SO_4_ at a liquid–solid ratio of 10:1 and stirred for 2 h at different reaction temperatures (20–80 °C). The leaching rate of Al_2_O_3_ was approximately 83% (Figure 4a). It remained basically unchanged with the increasing reaction temperature. Meanwhile, Fe was easier to leach than Al. The increasing reaction temperature promotes the activity of RM components, speeding up their reaction rate and diffusion. The viscosity of RM in the reaction system tends to be reduced, resulting in fewer substances adsorbed on its surface [28]. However, the leaching rate of Fe_2_O_3_ was basically the same at 50 °C and 65 °C. This may be caused by the large amount of leaching of interfering ions, such as calcium, magnesium, and silicon, which have varying degrees of impact on Fe ions. As illustrated in Figure 4c, the hematite crystalline structure in acid-leaching residue disappears with the increasing reaction temperature, whereas the gibbsite structure is unchanged. In addition, the peak value representing metallic elements increased significantly. Therefore, the increased reaction temperature contributed significantly to the leaching of Fe ions. The optimal reaction temperature was 80 °C.

### 3.3. Effect of the Initial H_2_SO_4_ Concentration on Al and Fe Leaching from Red Mud

At various concentrations of H_2_SO_4_ (20–80%), a reaction temperature of 20 °C for 2 h, and a liquid-to-liquid ratio of 10:1, SRM leaching of Al and Fe was determined. As shown in Figure 4b, the leaching rates of Al_2_O_3_ and Fe_2_O_3_ gradually increased with increasing concentrations of H_2_SO_4_. However, when the H_2_SO_4_ concentration was 60%, the exudation of Fe_2_O_3_ decreased slightly, which may because the large number of leaching of interfering ions leached have an impact on the leaching of Fe ions. The characteristic peaks of hematite and gibbsite weakened or disappeared (Figure 4d), indicating that both minerals were significantly dissolved with increasing H_2_SO_4_ concentrations. Increased H_2_SO_4_ concentration changed the Gibbs free energy [29], allowing the acid-leaching reaction to proceed more easily. Considering the cost of H_2_SO_4_, 50% concentrated H_2_SO_4_ was selected for further study.

### 3.4. Effect of the Reaction Time on Al and Fe Leaching from Red Mud

**Figure 5:**
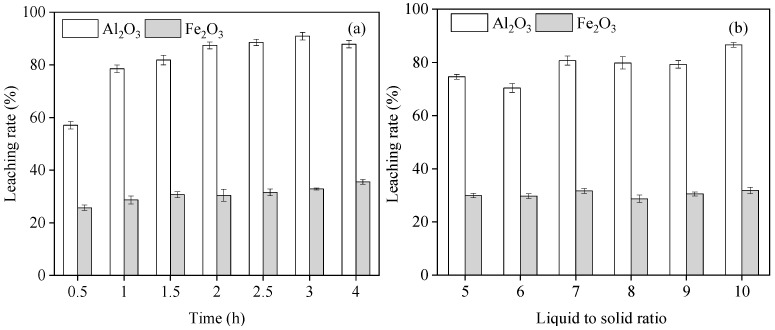
Effects of different treatment conditions on the SRM leaching rate of Al_2_O_3_ and Fe_2_O_3_: (**a**) reaction time at constant: H_2_SO_4_ concentration 20 wt%, liquid-to-liquid ratio 10:1 and reaction temperature 20 °C; (**b**) liquid-to-solid ratio at constant: H_2_SO_4_ concentration 20 wt% and reaction temperature 20 °C.

A mixture of SRM and 20% H_2_SO_4_ with a liquid-to-liquid ratio of 10:1 at 20 °C was used to determine the effects of the different reaction time (0.5–4 h) on the leaching rates of Al_2_O_3_ and Fe_2_O_3_. The leaching rate of Al_2_O_3_ increased rapidly in the first 1 h and thereafter stabilized, whereas that of Fe_2_O_3_ changed slightly with the increasing reaction time (Figure 5a). The average leaching rates of Al_2_O_3_ and Fe_2_O_3_ were approximately 81.74% and 30.79%, respectively. H_2_SO_4-_induced leaching of Al and Fe from RM was completed in a short time. Meanwhile, with the increased reaction time, flocs were formed, making the follow-up solid–liquid separation more difficult. Considering the leaching rate of Al_2_O_3_ and Fe_2_O_3_ and its influence on the subsequent filtration process, a suitable reaction time of 2 h was selected.

### 3.5. Effect of Liquid-to-Solid Ratio on Al and Fe Leaching from Red Mud

A mixture of SRM and 20% H_2_SO_4_ at 20 °C was used to determine the effects of different liquid–solid ratios (5:1–10:1) on the leaching of Al_2_O_3_ and Fe_2_O_3_ by RM. The leaching rates of Al_2_O_3_ fluctuated from 74.59% ± 0.93 to 86.63% ± 0.90 with an increasing liquid–solid ratio and the Fe_2_O_3_ kept slightly changing from 28.73% ± 1.44 to 31.66% ± 0.92 (Figure 5b). When the liquid-to-solid ratio was 10:1, the leaching rates of Al_2_O_3_ and Fe_2_O_3_ were 86.63 ± 0.90% and 31.86 ± 1.22%, respectively. Therefore, the best liquid–solid ratio was 10:1.

In summary, the optimal parameters for the RM acid leaching process were determined using single-factor experiments; that is, a sintering temperature of 700 °C, reaction temperature of 80 °C, H_2_SO_4_ concentration of 50%, reaction time of 2 h, and liquid-to-solid ratio of 10:1.

### 3.6. Characteristics of the Synthesized Flocculant

The acid leachate obtained under optimal conditions was used for flocculant (PAFS) synthesis. The PAFS production process comprised the oxidation of Fe ions, hydrolysis, and polymerization of Al and Fe ions. The relevant chemical reactions refer to the literature [15]:

(8) $$2Fe^{2+}+H_{2}O_{2}+2H^{+}\overset{\;\;}{\to }2Fe^{3+}+2H_{2}O$$

(9) $$Fe_{2}(SO_{4}{)}_{3}+nH_{2}O\overset{\;\;}{\to }Fe_{2}(OH{)}_{n}{{SO}_{4}^{2-}}_{3-n}+n{SO}_{4}^{2-}+nH^{+}$$

(10) $$Al(SO_{4}{)}_{3}+mH_{2}O\overset{\;\;}{\to }Al_{2}(OH{)}_{m}{{SO}_{4}^{2-}}_{3-m}+m{SO}_{4}^{2-}+mH^{+}$$

(11) $$Fe_{2}(OH{)}_{n}{{SO}_{4}^{2-}}_{3-n}+Al_{2}(OH{)}_{m}{{SO}_{4}^{2-}}_{3-m}\overset{\;\;}{\to }{Fe}_{2}{Al}_{2}{\left(OH\right)}_{n+m}{{SO}_{4}^{2-}}_{6-m-n}$$

**Figure 6:**
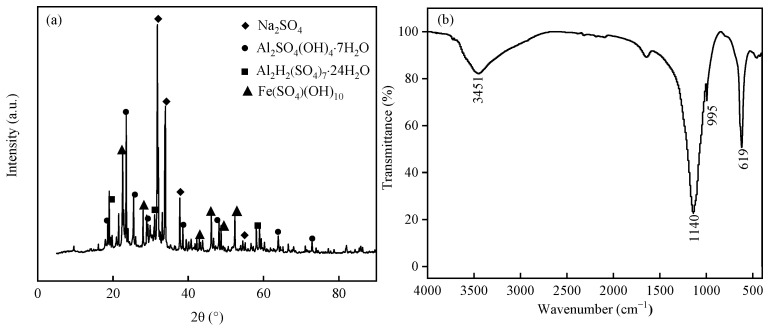
(**a**) X-ray diffractometry patterns of polymeric aluminum ferric sulfate. (**b**) Fourier transform infrared spectroscopy spectra of polymeric aluminum ferric sulfate.

The synthesized PAFS is a brownish-yellow solid flocculant with a density more than 1.80 g/cm^3^, pH value (1% aqueous solution) of 3.0–5.0, and salinity of more than 35%. As shown in Figure 6a, PAFS contained Al and Fe ions, indicating the occurrence of polymerization reactions to form Fe_2_(SO_4_)_3_ and Al_2_(SO_4_)_3_. The presence of several diffraction peaks with small peaks in the spectrum suggested that PAFS contains a few amorphous substances, resulting from the addition of Na^+^ during the polymerization process. Therefore, the produced PAFS is characterized by the co-existence of multiple crystalline phases.

Figure 6b presents a broad absorption peak around 3451 cm^−1^, which was attributed to the stretching vibration of -OH groups [30]. The wide peaks indicate that different atoms are connected to -OH. Combined with the chemical composition of the coagulant, it is speculated that there may be bonds such as Al-OH, Fe-OH, Si-OH and their internal H-OH adsorbing water molecules, resulting in polyhydroxyl polymers. The FTIR spectra showed medium and weak peaks at 1140 cm^−1^ and 995 cm^−1^, respectively (Figure 6b). Both were assigned to the asymmetric stretching vibration of Fe-O-Si or Al-O-Si [31]. Furthermore, the peak at 619 cm^−1^ was attributed to the bending vibration of Fe-OH and Al-OH [32]. The different absorption peaks indicate the polymerization of Fe^3+^ and Al^3+^ for PAFS production.

### 3.7. Turbidity and Phosphate Removal by the Synthesized Flocculant

#### 3.7.1. Effect of pH on Turbidity and Phosphate Removal

**Figure 7:**
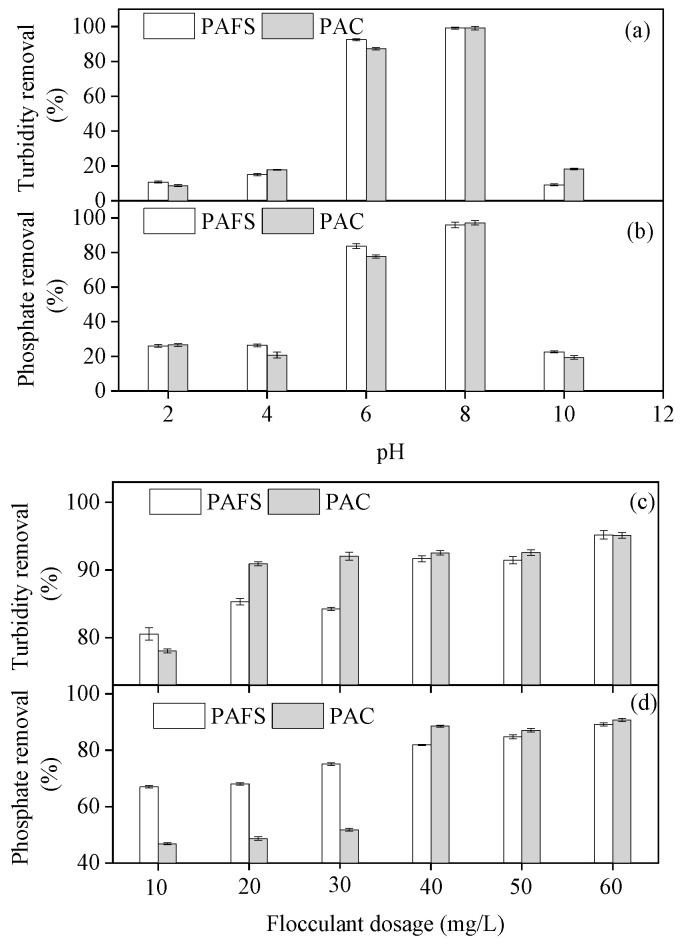
Effect of different dosage and initial pH of polymeric aluminum ferric sulphate and polyaluminum chloride on turbidity and phosphate removal efficiency. (**a**) Effect of initial pH on turbidity. (**b**) Effect of initial pH on phosphate. (**c**) Effect of dosage on turbidity. (**d**) Effect of dosage on phosphate.

The initial pH of the wastewater plays an important role in turbidity and phosphate removal as it affects the flocculant’s surface chemistry. Therefore, studying the effect of pH on turbidity and phosphorus removal is necessary to evaluate the flocculant’s performance [33]. In our study, PAC and PAFS displayed low efficiency at removing turbidity and phosphate under acidic and alkaline conditions (Figure 7a,b). Optimal turbidity and phosphate removal occurred at a pH value of 6.0–8.0, and the removal rates of turbidity by PAC and PAFS were 99.16 ± 0.95% and 99.22 ± 0.66%, respectively; the removal rates of phosphate by PAC and PAFS were 97.29 ± 1.16% and 95.98 ± 1.63%, respectively. When the pH is low, iron and aluminum salts mainly exist in the form of Fe^3+^, Al^3+^, Fe(OH)^2+^, and Al(OH)^2+^, and the phosphoric acid solution also mainly exists in the form of H_3_PO_4_. H_3_PO_4_ is not easily combined with the positively charged flocculant to form floc and is removed, so the phosphorus removal rate is low. When the pH = 6–8, phosphoric acid solution mainly exists in two forms, H_2_PO_4_^−^ and HPO_4_^2−^, and is easy to combine with the positively charged flocculant to form iron hydroxyphosphate and aluminum hydroxyphosphate precipitation [12], which continuously improves the phosphorus removal rate. As the pH further increases, OH^−^ replaces PO_4_^3−^ in iron hydroxyphosphate and aluminum hydroxyphosphate, forming hydroxide precipitation. At this time, phosphorus removal mainly relies on the adsorption of iron and aluminum hydroxide flocs, and the phosphorus removal rate decreases. In particular, some iron salts and aluminum salts generate soluble Fe(OH)_4_^−^ and Al(OH)_4_^−^, resulting in a further decrease in the phosphorus removal rate.

#### 3.7.2. Effect of the Synthesized Flocculant Dosage on Turbidity and Phosphate Removal

The effect of the PAFS dosage on turbidity and phosphate removal efficiency was investigated at a pH of 8.0. The turbidity and phosphate removal efficiency increased dramatically with increasing PAFS and PAC and reached stability. The turbidity and phosphate removal efficiency when the PAC dosage was 60 mg/L were 95.12 ± 0.42% and 90.75 ± 0.59%, respectively; when the PAFS dosage was 60 mg/L, the removal efficiency was 95.21 ± 0.64% and 89.17 ± 0.52%, respectively (Figure 7c,d). Therefore, the PAFS was an efficient flocculant for turbidity and phosphate removal. Previous studies have shown that the Zeta potential of flocculants increases from negative to positive with increasing dosage. When the Zeta potential approaches zero, the flocculant has the best performance of turbidity and phosphorus removal, which easily forms large flocs with a fast settling speed [34].

### 3.8. Properties of the Red Mud-Based Ceramsite

**Figure 8:**
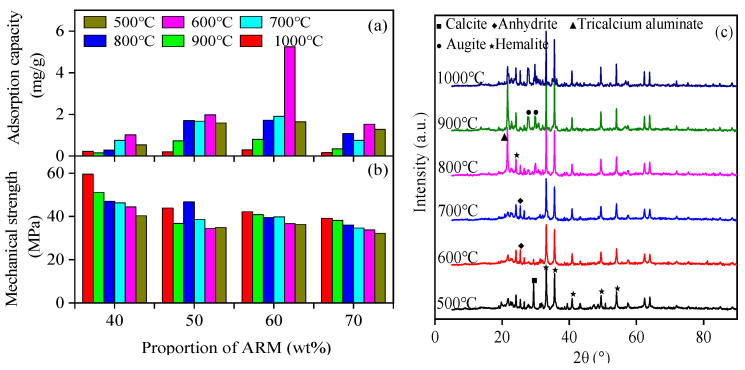
(**a**) Phosphate adsorption capacities of acid residue leaching-based ceramsite (ARMC). (**b**) Mechanical strength of acid residue leaching-based ceramsite. (**c**) X-ray diffractometry patterns of red mud granular adsorbents with 60% acid residue leaching at different sintering temperatures.

Preparation of high-strength ceramsite provided a novel method for efficient utilization of ARM. Not only can it reduce secondary pollution caused by ARM emissions, it can also be used as an adsorption material. Mechanical strength is a key indicator that affects its popularization and application [35]. The mechanical strength of the ARMC increased with the increasing calcination temperature. It reached a maximum at a sintering temperature of 1000 °C except for ARMC with 50% ARM (Figure 8). When ARM was 40%, the mechanical strength of ARMC was higher than the ARMC with other ARM ratios, with a maximum value of 59.55 MPa at 1000 °C. The ARMC adsorption capacity increased first, then decreased with the increasing sintering temperature. When ARM was 60%, the phosphate adsorption capacities of ARMC were optimal (5.25 mg/g) at a sintering temperature of 600 °C, and the mechanical strength was 36.63 MPa. To maximize the use of ARM, we chose ARMC with 60% of ARM sintering at 600 °C as the next research focus (Named: ARMC-60%-600 °C).

The XRD patterns of ARMC comprising 60% ARM at different sintering temperatures (Figure 8c) indicated that hematite was the main chemical constituent of the ARMC. The chemical composition of ARMC undergoes a transformation at 600 °C and 800 °C with the increased sintering temperature. When the sintering temperature rises to 600 °C, the amount of anhydrite increases with decreased calcite. Tricalcium aluminate and pyroxene peaked at a sintering temperature of more than 800 °C, indicating the formation of these two chemical components, and there were invisible anhydrite peaks simultaneously.

A previous study demonstrated that CaO, Fe_2_O_3_, and *η*, χ, γ-Al_2_O_3_ are the essential chemical components of ARMC when the sintering temperature is less than 800 °C [25]. The formation of Ca_3_Al_2_O_6_ from the reaction between CaO with Al_2_O_3_ and Fe_2_O_3_ decreased with the increasing sintering temperature, which is consistent with the results of XRD analysis of ARMC. The formation of Ca_3_Al_2_O_6_ and Ca (Mg, Fe, Al) (Si, Al)_2_O_6_ can strengthen the ARMC. Therefore, the increased sintering temperature significantly improves the strengthening effect, and the structure is more stable, especially above 900 °C.

The surface of ARMC-60%-600 °C was rougher and had more pore structures than others, which was confirmed by the results of the BET analysis of ARMC with different mass ratios at 600 °C (Table 3). The pore volume of ARMC-60%-600 °C was 0.167 cm^3^/g, which is larger than 0.149 cm^3^/g, 0.128 cm^3^/g, and 0.025 cm^3^/g for ARMC-70%-600 °C, ARMC-50%-600 °C, and ARMC-40%-600 °C, respectively. A greater pore volume indicates more BET surface areas for phosphate adsorption.

**Figure 9:**
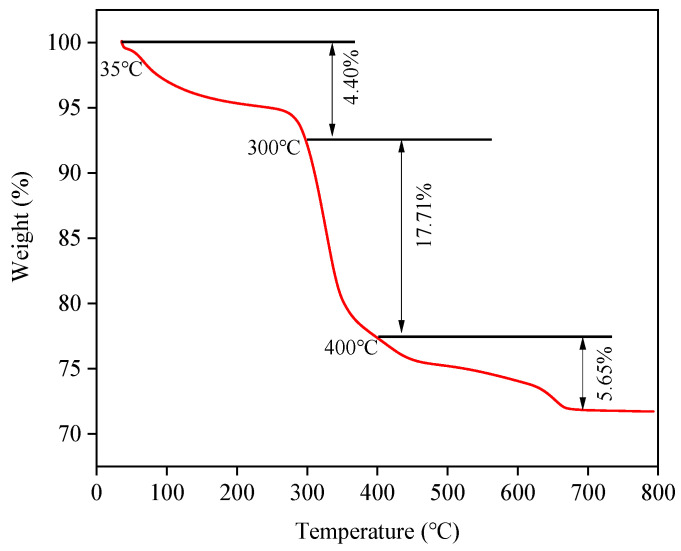
Thermogravimetric curve of acid leaching residue-based ceramsite-60%-600 °C at a heating rate of 5 °C/min.

Moreover, ARMC-60%-600 °C showed different degrees of weight loss with increased temperature, which could be divided into three stages according to the weight loss curve (Figure 9). The initial weight loss was 4.40% at 35–300 °C, which can be attributed to the volatilization of adsorbed water on the surface. The second-stage weight loss was 17.71% at 300–400 °C, mainly due to the removal of water in the internal mineral crystals, accompanied by the carbonization of organic matter [24]. When the temperature rises above 400 °C, the third weight loss is 5.65%, which finally tends to be stable due to the decomposition of chemicals (such as releasing a small amount of gas from carbonate decomposition) [36]. Owing to ARMC-60%-600 °C thermal instability, it is reasonable to choose 400 °C as the preheating temperature, which allows the removal of surface-adsorbed and constituent water from ARMC-60%-600 °C, while ensuring that the effective carbonization of organic matter and chemical substances will not be decomposed in advance at a high temperature.

**Figure 10:**
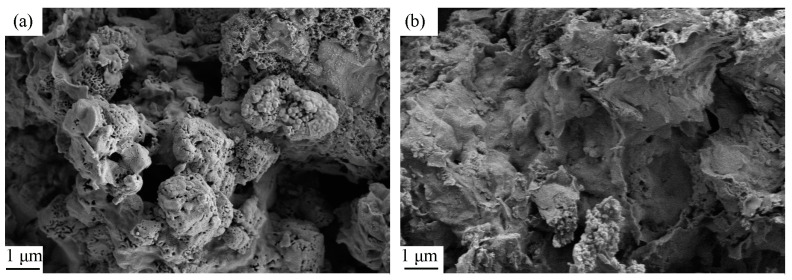
The scanning electron microscope images of acid leaching residue-based ceramsite (ARMC)-60%-600 °C (**a**) before phosphate adsorption, and (**b**) after phosphate adsorption.

**Figure 11:**
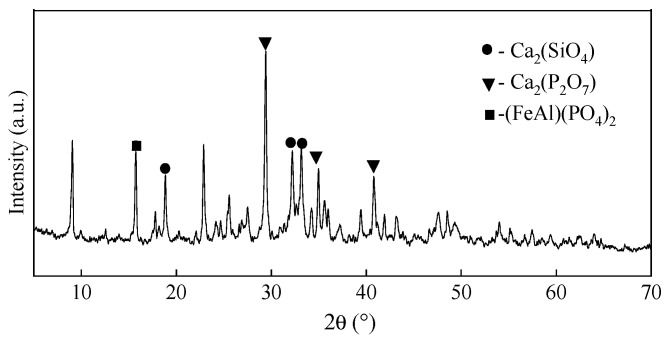
X-ray diffractometry patterns of acid leaching residue-based ceramsite (ARMC)-60%-600 °C after phosphate adsorption.

The SEM before and after phosphate adsorption at ARMC-60%-600 °C showed that the appearance of ARMC-60%-600 °C before adsorption is rough, and the pore distribution on the surface is relatively uniform (Figure 10), and is caused by the gas produced in the sintering process. After adsorption, the pores on the surface seem to be occupied. Furthermore, no evidence exists to show that the surface of ARMC-60%-600 °C was eroded or collapsed, indicating that RM granular adsorbents can resist water erosion and the loss of effective adsorption components. After phosphorus adsorption, ARMC particles formed a phosphorus-containing phase of Ca_2_(P_2_O_7_) (Figure 11). In addition, there was also a crystal phase of (FeAl)(PO_4_)_2_, indicating that phosphorus was adsorbed successfully.

## 4. Discussions

Previous research suggested that sintering of RM contributed to the leaching of Fe and Al ions [37] and had been used for Fe and Al extraction [18,38,39]. Recently, Feng and Yang (2018) [40] have reported that the composition of Bayer RM from the drying process was Fe_2_O_3_ (7.69 ± 0.09%), Al_2_O_3_ (20.52 ± 0.09%), SiO_2_ (22.47 ± 0.16%), CaO (18.42 ± 0.09%), and TiO_2_ (4.92 ± 0.02%), the corresponding results for the sintering RM were Fe_2_O_3_ (8.50 ± 0.09%), Al_2_O_3_ (7.28 ± 0.07%), SiO_2_ (24.02 ± 0.18%), CaO (44.00 ± 0.2%), and TiO_2_ (4.17 ± 0.08%), and the sintering RM showed much higher levels of CaO but low levels of Al_2_O_3_ as compared to the RM; similar trends have also been reported by Wang and Liu (2012) [41], which indicates that a different sintering temperature has a significant impact on the leaching of Fe and Al. In this study, RM sintering at 600 °C had a positive effect on the leaching of iron and aluminum.

The composite copolymerization of Al-salt and Fe-salt is not a simple mechanical mixture, but a new type of composite water-soluble polymer formed by physical and chemical reactions. The flocculation mechanism of PAFS is dominated by electric neutralization, with auxiliary effects of adsorption bridging and net capture [42]. Pan et al. (2012) [43] suggested that there are hydroxyl bridges between irons and between aluminum, and the strong association between iron, aluminum and hydroxyl groups, so as to achieve PAFS preparation. When PAFS is added to the aqueous solution, it will hydrolyze to form a multinuclear hydroxyl complex micelle bond and a large number of Al^3+^ and Fe^3+^, and then connect with each other to form a ring structure, and finally crisscross each other to form a three-dimensional network structure, which has a strong adsorption capacity and can adsorb smaller organic compounds [15]. As the dosage of the flocculant gradually increases, it begins to settle. During the settling process, it continues to adsorb small particles in the solution, promoting a continuous increase in the volume of flocculent particles and an acceleration in the settling speed, thus achieving the ideal flocculation effect.

The residue after leaching still contains a certain amount of Al, Fe, Na, and Ca, which can cause secondary pollution to the environment if discharged [44]. The production of ARMC not only reuses the residue, but also provides a new possibility for the selection of phosphorus removal substrate in constructed wetland. In this study, the ARMC has a strength of 36.63 MPa at a sintering temperature of 600 °C, which is higher than high-strength ceramsite of 21.01 MPa was prepared from red mud, fly ash, and bentonite by Mi et al. (2021) [45] and 3.3 MPa by Ma and Li (2013) [46]. Moreover, the maximum adsorption capacity of ARMC for phosphate was 5.25 mg/g at a sintering temperature of 600 °C, which is higher than the red mud-based ceramic medium of 0.42 mg/g produced by Shabnam et al. (2019) [21]. Therefore, ARMC is an efficient phosphorus adsorption material, which can be used in the advanced treatment of sewage in constructed wetland.

## 5. Conclusions

This study showed a novel method of synthesizing flocculant (PAFS) from RM. Moreover, the acidified RM residue (ARM) was used to synthesize ceramsite (ARMC). We determined that the rawing RM sintering (SRM) at 700 °C presented the optimal conditions for Al and Fe ions leaching. Additionally, it showed that the concentration of Fe and Al was further leached after the SRM was added into 50% concentrated sulfuric acid shaking with a liquid–solid ratio of 10:1 at 80 °C for 2 h. The synthesized PAFS was a solid brownish-yellow flocculant with a density of more than 1.80 g/cm^3^, pH value of 3.0–5.0, and salinity of more than 35%. When the initial pH ranged from 2.0 to 10.0, the turbidity and phosphate removal efficiency by PAFS increased first and then decreased, with the best removal efficiency at pH = 8. At a PAFS dosage of 60 mg/L, the turbidity and phosphate removal efficiency by PAFS increased to 95.21 ± 0.64% and 89.17 ± 0.52%, respectively. To maximize the use of ARM, taking into account the phosphate adsorption capacity and mechanical strength of ARMC, we chose the ARM-based ceramsite with 60% of ARM and sintering at 600 °C as the best ceramsite, while the BET surface area was 56.16 m^2^/g and pore volume was 0.167 cm^3^/g.

## Figures and Tables

**Table 1:** Chemical compositions of RM (wt%).

Components	Na_2_O	MgO	Al_2_O_3_	SiO_2_	CaO	TiO_2_	Fe_2_O_3_	K_2_O	Others
Mass percentage	10.77	1.12	23.11	20.25	11.50	4.01	26.96	0.65	1.63

**Table 2:** The quality of wastewater used for the evaluation of performance in flocculation.

Water Quality Index	COD (mg/L)	TP (mg/L)	TN (mg/L)	NH_3_-N (mg/L)	pH
Concentration	240.45	2.98	40.78	55.32	7.32

**Table 3:** Surface area and pore volume of ARMC.

Parameters	ARMC-70%-600 °C	ARMC-60%-600 °C	ARMC-50%-600 °C	ARMC-40%-600 °C
BET surface area (m^2^/g)	40.62	56.16	25.24	4.79
Pore volume (cm^3^/g)	0.149	0.167	0.128	0.025

## Data Availability

Data are contained within the article.
